# Incidence, Prognostic Factors, and Survival Trend in Pineal Gland Tumors: A Population-Based Analysis

**DOI:** 10.3389/fonc.2021.780173

**Published:** 2021-11-19

**Authors:** Huy Gia Vuong, Tam N. M. Ngo, Ian F. Dunn

**Affiliations:** ^1^ Department of Neurosurgery, Oklahoma University Health Sciences Center, Oklahoma City, OK, United States; ^2^ Department of Pathology, Oklahoma University Health Sciences Center, Oklahoma City, OK, United States; ^3^ Faculty of Medicine, Pham Ngoc Thach University of Medicine, Ho Chi Minh City, Vietnam

**Keywords:** pineal gland, brain tumor, germ cell tumor, pineal parenchymal tumor, pineoblastoma, glioma, survival trend

## Abstract

**Introduction:**

Pineal gland tumors are exceedingly rare and account for 0.4-1.0% of brain neoplasms. Their rarity has confounded a clear understanding of the prognostic factors and standards of care for these neoplasms. In this study, we aimed to investigate the incidence, prognostic indicators, and survival trend of tumors emanating from the pineal gland.

**Methods:**

We accessed the Surveillance, Epidemiology, End Results (SEER) Program for pineal gland tumors from 1975-2016. A multivariate Cox regression model was used to investigate the impact of clinicopathological parameters on all-cause mortality. For survival trend analysis, we employed the Kaplan Meier curve and pairwise comparisons to examine the trend.

**Results:**

We found 1,792 and 310,003 pineal gland and brain neoplasms during 1975-2016 resulting in an incidence of 0.6%. In the multivariate Cox proportional hazards model, older age, male gender, non-germ cell tumor, and receipt of chemotherapy were significantly associated with poor survival (*p* < 0.001). The extent of resection and radiotherapy administration did not produce survival advantages. Our result also highlighted an increased survival of pineal gland tumors over the years.

**Conclusion:**

Our study investigated the prognostic factors that influenced survival in patients with pineal gland tumors. Chemotherapy use adversely affected patient outcomes and should be considered carefully in specific circumstances to avoid its harmful effects. These findings provide important evidence to improve current standards of care for this rare group of tumors. The survival of pineal tumors has improved over time reflecting improvements in current practice.

## Introduction

Pineal gland tumors are very rare brain tumors that account for less than 1% of all central nervous system (CNS) tumors in the United States (1, 2). These neoplasms are typically seen in young males and present with symptoms of obstructive hydrocephalus and/or compression of the tectum (1, 3). The 5- and 10-year survival rates of all pineal gland tumors are 75.9% and 71.5%, respectively (2). Adolescent and young adult patients have the best prognosis while the pediatric group (age 0-14) has the worst outcome (2). Histologically, pineal tumors are classically divided into germ cell tumors (GCT), pineal parenchymal tumors (PPT), gliomas, and others (3).

GCTs are the most common pineal gland neoplasms, accounting for about 60% of pineal tumors (1, 2). Based on the 2016 World Health Organization (WHO) classification, central nervous system (CNS) GCTs include germinoma, embryonal carcinoma, yolk sac tumor, choriocarcinoma, mature/immature teratoma, and mixed GCT. Among them, germinoma is the most frequent subtype accounting for 76% of CNS GCT (1). GCTs are typically sensitive to radiotherapy and are associated with a superior outcome.

The second most common pineal gland tumor is PPT and is half as common as the GCT (1). PPTs include pineocytoma, PPT of indeterminate differentiation, pineoblastoma, and papillary tumor of the pineal region (3). Pineal glioma only constitutes 2.8% of all glial neoplasms but they are the third most common pineal gland tumor (2). Other CNS tumors that can arise from the pineal gland stroma are atypical teratoid/rhabdoid tumor (ATRT), ependymoma, ganglioma, and meningioma.

Because of the rarity, it is difficult to perform large-sized cohort studies to investigate the prognostic factors of pineal gland neoplasms. In this population-based analysis, we also aimed to perform trend analyses on patient survival over time.

## Materials and Methods

We searched for pineal gland tumor data (primary site of C75.3) in the Surveillance, Epidemiology, End Results (SEER) 18 registries custom database from 1975-2016 without age restriction. Patients with autopsy or death certificate only were removed from the analyses. We also excluded cases with missing information for demographic (age, gender, and race), treatment fields (surgery, radiotherapy, and chemotherapy), and follow-up data. Unclassified intracranial or intraspinal neoplasms with histology codes of 8000, 8001, and 8002 were also excluded because they do not indicate specific histology types. Primitive neuro-ectodermal tumor was reclassified as pineoblastoma because studies have shown that they are clinically, histologically, and molecularly similar (4, 5). In addition, cases with the histology code of “pinealoma”, a term used in previous WHO classifications, were also omitted because they were nonspecifically used for both GCTs and PPTs. Histologically, pineal neoplasms were categorized into GCT, PPT, gliomas, and others. Applying these selection criteria resulted in a final cohort of 1,166 pineal tumors diagnosed from 1998-2016 (1998 is the first year when treatment data were tabulated). The following variables were extracted from the SEER database: patient ID, age at diagnosis, gender, race, year of diagnosis, histologic diagnosis, WHO grade, tumor size, distant metastasis at presentation, extent of resection, radiotherapy, chemotherapy, all-cause mortality status and survival time.

We tested the associations of various clinical and treatment parameters with the extent of resection using the Pearson’s Chi-square test and Fisher’s exact test for categorical variables, and the Mann-Whitney test and Student t-test for continuous variables, if applicable. For time-to-event data, we utilized the log-rank test and multivariate Cox proportional hazards model to analyze the impact of different clinical parameters on all-cause mortality. Proportionality assumptions of the Cox regression models were assessed by log-log survival curves and with the use of Schoenfeld residuals. The deviance residuals and the dfbeta values were used to examine influential observations. Hazard ratios (HR) are presented as mean and 95% confidence intervals (CI). We considered a statistically significant result if the *p-*value of less than 0.05. Statistical analyses were conducted by SPSS version 20 (IBM, New York, NY) and R software, version 4.0.3 (The R Foundation, Vienna, Austria)

For survival trend analysis, we divided cases diagnosed between 1975-2016 into four groups as followed: 1975-1984, 1985-1994, 1995-2004, and 2005-2016. We examined patient survival between these periods using pairwise comparison over strata.

## Results

From 1975-2016, the total number of brain tumors in the SEER database was 310,003 cases. During this period, we found 1,792 pineal gland tumors resulting in an incidence of 0.6%.

### Characteristics of Pineal Gland Tumors

After deleting 626 cases with missing data (46 cases with histologic diagnosis of pinealoma, 209 cases with unclassified histologies, 60 cases diagnosed at autopsy or with death certificate only, and 311 cases with missing treatment data), we obtained a total number of 1,166 pineal tumors for data analyses. Two-third of patients were males and the median age of diagnosis was 19 years of age (range, 0-94). Histologically, GCT and PPT comprised 45.6% and 44.9% of pineal gland neoplasms, respectively, followed by glioma (5.3%), and ATRT (0.8%). The median tumor size was 26mm and distant metastasis at presentation was observed in 6.3% of cases. Surgically, gross total resection (GTR) was only achieved in a subset of pineal tumors (8.5%). The rates of radiotherapy and chemotherapy administration were 63.1% and 49.4%, respectively.

### Prognostic Factors of All-Cause Mortality

**Table 1** shows the associations of demographic and clinical parameters with the extent of resection. Biopsy was more likely applied for GCT while non-GCT underwent STR and GTR (*p* < 0.001). Additionally, GTR was more frequently achieved in younger patients (median age of 15 years) as compared to biopsy and STR (*p* < 0.001). We also found a significant association of tumor size with the extent of resection; specifically, biopsied tumors had a smaller size as compared to resected tumors. Also, biopsied tumors were associated with a significantly higher rate of distant metastasis at presentation. There were no significant associations of the extent of resections with other parameters including gender, race, receipt of radiotherapy/chemotherapy, and patient mortality.

**Table 1:** The associations of various parameters with the extent of resection.

Parameters	All	Biopsy	STR	GTR	p-value*
Patient no. (%)	1166 (100.0)	541 (46.3)	526 (45.3)	99 (8.4)	
Age, median (IQR) (years)	19 (12-36)	19 (14-37)	20 (12-37)	15 (8-24)	**<0.001**
Gender, no. (%)					0.191
Female	388 (33.3)	166 (30.7)	189 (35.9)	33 (33.3)	
Male	778 (66.7)	375 (69.3)	337 (64.1)	66 (66.7)	
Race, no. (%)					0.730
White	883 (75.7)	413 (76.3)	393 (74.7)	77 (77.8)	
Non-white	283 (24.3)	128 (23.7)	133 (25.3)	22 (22.2)	
Histology, no. (%)					**<0.001**
GCT	532 (45.6)	304 (56.2)	187 (35.6)	41 (41.4)	
PPT	524 (44.9)	190 (35.1)	286 (54.4)	48 (48.5)	
Glioma	62 (5.3)	24 (4.4)	34 (6.5)	4 (4.0)	
Others	48 (4.1)	23 (4.3)	19 (3.6)	6 (6.1)	
Tumor size, median (IQR) (mm)	26 (20-34)	24 (17-31)	28 (21-36)	30 (23-34)	**<0.001**
DM at presentation, no. (%)	57 (6.8)	38 (9.9)	14 (3.6)	5 (7.8)	**0.002**
Radiotherapy, no. (%)	736 (63.1)	328 (60.6)	345 (65.6)	63 (63.6)	0.243
Chemotherapy, no. (%)	576 (49.4)	277 (51.2)	245 (46.6)	54 (54.5)	0.180
Patient mortality, no. (%)	251 (21.5)	109 (20.1)	114 (21.7)	28 (28.3)	0.193

DM, distant metastasis; GCT, germ cell tumor; GTR, gross total resection; IQR, interquartile range; PPT, pineal parenchymal tumor; STR, subtotal resection.

*bold value indicates a statistically significant result.

Multivariate Cox regression model demonstrated that older age, male gender, non-GCT histology, and chemotherapy use were prognostic factors that negatively influence all-cause survival (**Table 2**). The greater extent of resection and radiation receipt added no benefits to patient survival.

**Table 2:** Multivariate Cox proportional hazards regression for all-cause mortality of pineal gland tumors.

Variable		Hazard Ratio (95% CI)	*p*-value*
Age	Per year increase	1.019 (1.012-1.026)	**<0.001**
Gender	Female	Reference	
	Male	1.609 (1.215-2.130)	**0.001**
Race	Non-white	Reference	
	White	0.932 (0.701-1.238)	0.627
Histology	GCT	Reference	
	Non-GCT	3.948 (2.808-5.549)	**<0.001**
Resection	None/Biopsy	Reference	
	STR	0.973 (0.741-1.278)	0.844
	GTR	1.255 (0.818-1.924)	0.298
Radiation	No	Reference	
	Yes	0.781 (0.585-1.043)	0.094
Chemotherapy	No	Reference	
	Yes	2.593 (1.876-3.584)	**<0.001**

CI, confidence interval; GCT, germ cell tumor; GTR, gross total resection; STR, subtotal resection.

*bold value indicates a statistically significant result.

Stratified by tumor histology, older age, male gender, and use of chemotherapy were negative prognostic factors of non-GCT of the pineal gland; whereas radiotherapy administration brought survival advantages to these patients (**Table S1**). In GCT, white patients were associated with a superior outcome in comparison to the non-white population whereas older age remained a poor prognostic indicator. The extent of resection had no survival impact regardless of the tumor pathological diagnoses. Chemotherapy administration was only associated with an inferior outcome in non-GCTs (**Table S1**), and this association was not seen in GCTs (**Table S2**).

### Survival Trends of Pineal Gland Tumors

**Figure 1:**
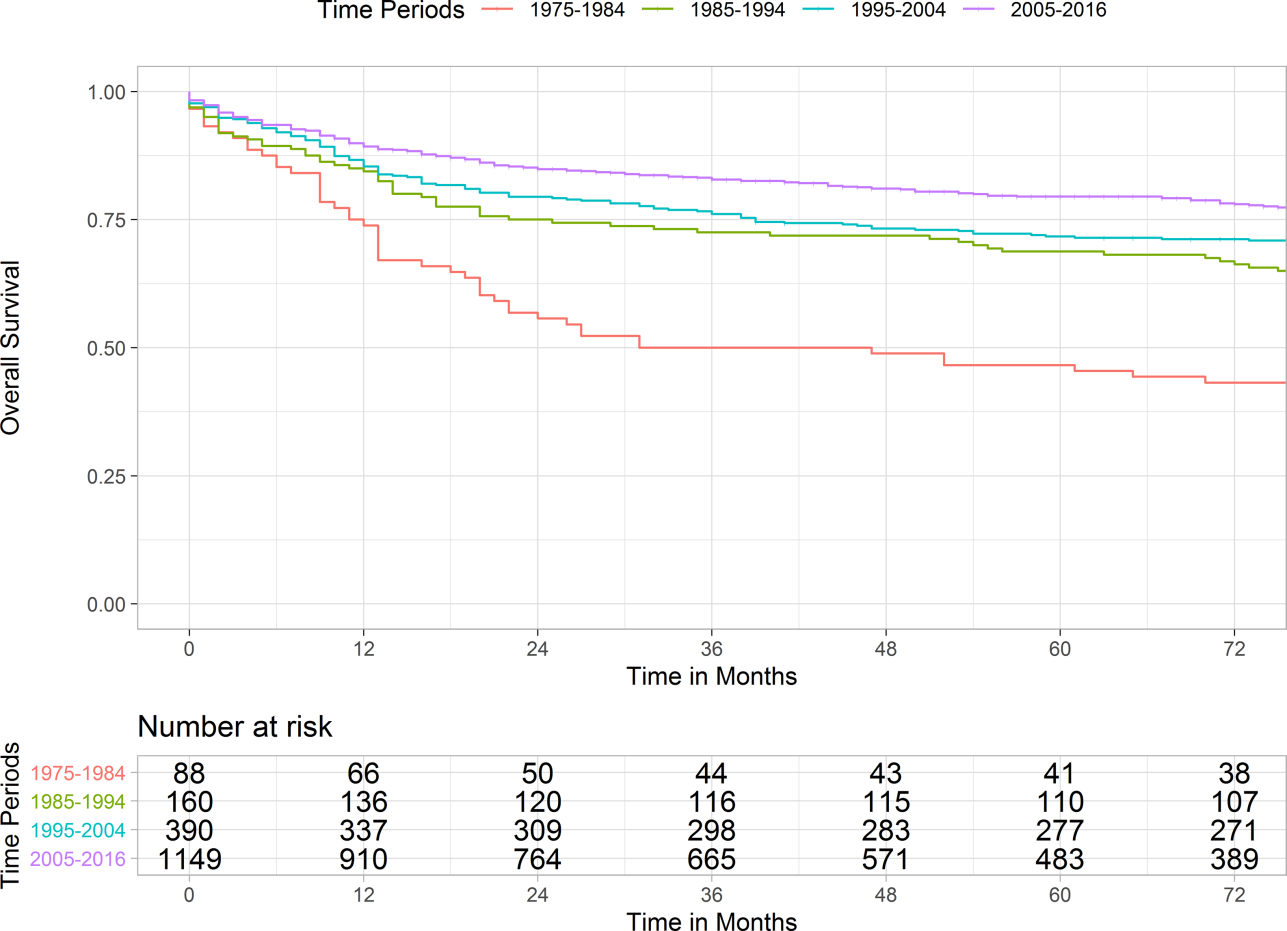
Kaplan Meier curve illustrating the survivals of pineal gland tumors over four periods. Pairwise comparison results were as followed: 1975-1984 *versus* 1985-1994, *p*-value < 0.001; 1985-1994 *versus* 1995-2004, *p*-value = 0.860; 1995-2004 *vs.* 2005-2016, *p*-value = 0.003.

Kaplan-Meier curve and pairwise comparisons demonstrated that patients diagnosed between 1975-1984 conferred the worst prognosis as compared with other periods. Patients with the year of diagnosis of 1985-1994 and 1995-2004 had a similar outcome (*p* = 0.860). Cases diagnosed between 2005-2016 had a significantly longer survival in comparison with the remaining periods (**Figure 1** and **Table S3**). **Table 3** shows the 1-, 3-, and 5-year survival rates of each period.

**Table 3:** The 1-, 3-, and 5-year survival rates of pineal gland tumors in four periods.

Periods	Time (months)	Number at Risk	Number of Events	Survival rate (%)	95% Confidence Interval	
					Lower	Upper
2005-2016	12	910	120	89.0 %	87.2 %	90.9 %
	36	665	59	82.6 %	80.2 %	84.9 %
	60	483	23	79.3 %	76.7 %	82.0 %
1995-2004	12	337	57	85.4 %	81.9 %	88.9 %
	36	298	36	76.1 %	72.0 %	80.5 %
	60	277	17	71.7 %	67.4 %	76.3 %
1985-1994	12	136	25	84.4 %	78.9 %	90.2 %
	36	116	19	72.5 %	65.9 %	79.8 %
	60	110	6	68.8 %	61.9 %	76.3 %
1975-1984	12	66	25	72.2 %	63.5 %	82.1 %
	36	44	21	48.9 %	39.6 %	60.4 %
	60	41	3	45.6 %	36.3 %	57.1 %

## Discussion

From our study, the estimated incidence of pineal region tumors among brain neoplasms was 0.6%, which is in line with previous reports (1, 2, 6, 7). The principal cells of the pineal gland are the pineal parenchymal cells which are the origin of PPTs. There are other cell types located adjacent to the gland resulting in diverse pathologies seen in this small endocrine gland such as GCTs, gliomas, embryonal tumors, or gangliomas. Our findings confirmed that GCTs have a better prognosis than other pineal neoplasms (1, 8), affirming that histology is a crucial factor in determining outcome of these tumors, rather than tumor grade (9, 10). Pineal gland tumors have the potential for distant spread through cerebrospinal fluid (CSF) seeding and drop metastasis. Patient prognosis was dramatically affected by CSF tumor spread at presentation. It is critical to screen the full neuroaxis in all patients with pineal region tumors.

There are different surgical approaches to the pineal region depending on the specific tumor location and surgeon preference. They include paramedian/midline infratentorial supracerebellar resection (11, 12), occipital transtentorial resection (13), and endoscopic tumor resection or biopsy (14). Our study also showed that surgical approaches are dependent on tumor pathology, for which biopsy was more preferable for GCT. There is debate as to what extent of resection is adequate to treat pineal region tumors (10, 15–17). Our results suggested that surgical approaches did not influence patient outcomes regardless of patient demographic, tumor histology, and other treatment modalities. These findings may help to justify safer and minimally invasive surgical techniques for pathological confirmation only and avoid the risk of overtreatment in select cases. Complications and morbidities following pineal region surgery are not uncommon such as CSF leak, meningitis, and hemorrhage (15).

In this population-based cohort, GCTs and PPTs were the most frequent neoplasms with an equal proportion. Germinomas are typically radiosensitive (18, 19) while this treatment modality seems to be less effective in PPTs (20–22). However, our analyses demonstrated that radiation treatment actually added survival benefits to non-GCT patients while no survival advantages were observed in radiated versus non-radiated GCTs. In the most recent SEER report on pineal gland tumor in 2009, radiotherapy receipt was an independent factor that had a positive impact on survival (1). Of note, several important parameters were not included in the survival modeling including race and chemotherapy. In addition, the number of included patients in this study was nearly as twice as the previous report which could improve the statistical adjustment. After including chemotherapy in the multivariate analysis, we demonstrated that this treatment modality did not confer a survival advantage and even decreased patient survival, particularly in non-GCT patients. Selection bias would be controlled for in this population analysis, but the use of chemotherapy in tumors already associated with poor survival is a consideration. Therefore, the use of chemotherapy should be cautiously considered in specific circumstances such as an alternative treatment for cranial radiation in very young children to avoid neuropsychological dysfunction or growth delay.

This study demonstrated several important prognostic factors to predict pineal gland tumor outcomes. Younger age at diagnosis, female gender, GCT histology, and no chemotherapy use were indicators for an improved prognosis. We observed improved patient survival of pineal gland tumors over the years. Patients diagnosed in 1975-1984, 1985-2004, and 2005-2016 had a steadily increased outcome. The uniformly improved patient survival of pineal tumors might be a consequence of major health care adjustments and technological advances. Advances in serology, imaging, and pathology enable earlier and more accurate diagnosis and tumor staging while improvements in surgical techniques, radiation, and chemotherapy are key changes leading to more effective treatment and management of patients.

This study may provide applicable evidence on pineal gland tumors which may help improve patient management and appropriately tailor treatment decisions. However, several limitations need to be considered. Firstly, data on tumor progression and recurrence were not available in the SEER dataset which could affect our analyses. Next, data on WHO grades are missing in a number of cases so we did not include this parameter in the survival analysis. Other important factors such as patient symptoms, performance status, radiation dosages, and chemotherapy regimen details that could affect patient prognosis were not reported. Lastly, this study is subject to the myriad constraints of using a national population-based database, including lack of individualized follow-up and in-depth scrutiny of extreme short-term or long-term survivors.

In conclusion, this population-based study outlined the important prognostic significance of several demographic/clinical parameters in patients with pineal gland tumors. Surprisingly, the extent of resection and radiation administration did not affect all-cause mortality while the use of chemotherapy was negatively associated with patient survival. These findings highlight a potential role for careful consideration in deploying these modalities among these patients. Our results also demonstrated improved survival of patients with pineal gland neoplasms over the years.

## Data Availability Statement

The original contributions presented in the study are included in the article/**Supplementary Material**. Further inquiries can be directed to the corresponding author.

## Author Contributions

HV: conceptualization, data curation, formal analysis, investigation, methodology, project administration, software, validation, writing-review, and editing. TN: data curation, formal analysis, investigation, methodology, writing-review. ID: project administration, validation, writing-review, editing, and supervision. All authors have read and approved the manuscript.

## Conflict of Interest

The authors declare that the research was conducted in the absence of any commercial or financial relationships that could be construed as a potential conflict of interest.

## Publisher’s Note

All claims expressed in this article are solely those of the authors and do not necessarily represent those of their affiliated organizations, or those of the publisher, the editors and the reviewers. Any product that may be evaluated in this article, or claim that may be made by its manufacturer, is not guaranteed or endorsed by the publisher.
