# Supplementary material for: Incidence, Prognostic Factors, and Survival Trend in Pineal Gland Tumors: A Population-Based Analysis
Source: Front Oncol. 2021 Nov 19;11:780173. doi: 10.3389/fonc.2021.780173 (PMC8639690; doi:10.3389/fonc.2021.780173)
Supplement: Supplementary file 1 [file Table_1.docx]

**Incidence, prognostic factors, and survival trend of pineal gland tumors: a population-based analysis**

Vuong et al. (Supplementary data)

Table S1. Multivariate Cox regression analysis for overall survival of pineal non-GCTs

| **Variable** | | **Hazard Ratio (95% CI)** | **p-value*** |
| --- | --- | --- | --- |
| Age | Per year increase | 1.020 (1.012-1.028) | **<0.001** |
| Gender | Female | Reference |  |
|  | Male | 1.780 (1.331-2.380) | **<0.001** |
| Race | Non-white | Reference |  |
|  | White | 1.141 (0.810-1.607) | 0.45 |
| Resection | Biopsy | Reference |  |
|  | STR | 0.863 (0.631-1.179) | 0.354 |
|  | GTR | 1.184 (0.738-1.900) | 0.483 |
| Radiation | No | Reference |  |
|  | Yes | 0.678 (0.485-0.948) | **0.023** |
| Chemotherapy | No | Reference |  |
|  | Yes | 3.621 (2.479-5.287) | **<0.001** |

Abbreviations: CI, confidence interval; GCT, germ cell tumor; GTR, gross total resection; STR, subtotal resection

*, bold value indicates a statistically significant result

Table S2. Multivariate Cox regression analysis for overall survival of pineal GCTs

| **Variable** | | **Hazard Ratio (95% CI)** | **p-value*** |
| --- | --- | --- | --- |
| Age | Per year increase | 1.034 (1.013-1.057) | **0.002** |
| Gender | Female | Reference |  |
|  | Male | 0.540 (0.239-1.218) | 0.138 |
| Race | Non-white | Reference |  |
|  | White | 0.536 (0.317-0.907) | **0.02** |
| Resection | Biopsy | Reference |  |
|  | STR | 1.369 (0.802-2.338) | 0.25 |
|  | GTR | 1.055 (0.370-3.006) | 0.921 |
| Radiation | No | Reference |  |
|  | Yes | 1.039 (0.541-1.994) | 0.908 |
| Chemotherapy | No | Reference |  |
|  | Yes | 1.123 (0.643-1.959) | 0.684 |

Abbreviations: CI, confidence interval; GCT, germ cell tumor; GTR, gross total resection; STR, subtotal resection

*, bold value indicates a statistically significant result

Table S3. Pairwise comparison for the survival difference across the four groups

| Variable | *p* value | | | | |
| --- | --- | --- | --- | --- | --- |
|  | Univariate correction | Shaffer | Bonferroni | Hochberg | Holm |
| Group 2- Group 1 | < 0.001 | < 0.001 | < 0.001 | < 0.001 | < 0.001 |
| Group 3- Group 1 | < 0.001 | < 0.001 | < 0.001 | < 0.001 | < 0.001 |
| Group 4- Group 1 | < 0.001 | < 0.001 | < 0.001 | < 0.001 | < 0.001 |
| Group 3- Group 2 | 0.86 | 0.86 | 0.99 | 0.86 | 0.86 |
| Group 4- Group 2 | 0.01 | 0.01 | 0.08 | 0.03 | 0.03 |
| Group 4- Group 3 | 0.003 | 0.009 | 0.018 | 0.009 | 0.009 |
| Group 1: 1975-1984; Group 2: 1985-1994; Group 3: 1995-2004; Group 4: 2005-2016 | | | | | |
